# Characterization of novel 3D‐printed metal shielding for brachytherapy applicators

**DOI:** 10.1002/acm2.14541

**Published:** 2024-10-12

**Authors:** K. Maiti McGrath, Krista Chytyk‐Praznik, Amanda Cherpak

**Affiliations:** ^1^ Department of Physics and Atmospheric Science Dalhousie University Halifax Canada; ^2^ Department of Radiation Oncology Dalhousie University Halifax Canada; ^3^ QE2 Cancer Centre Nova Scotia Health Halifax Canada

**Keywords:** 3D printed applicators, gynecological brachytherapy, shielded applicators

## Abstract

**Purpose:**

To characterize 3D‐printed stainless steel metal samples in the presence of an Iridium‐192 source for organ‐at‐risk sparing in gynecologic brachytherapy.

**Methods:**

Samples of 3D‐printed stainless steel (5.5 × 5.5 cm^2^, thickness range 1–5 mm) were embedded in a solid water phantom at varying distances from source catheters. An Ir‐192 brachytherapy source was passed through the phantom and the dose was measured using EBT3 Gafchromic film. The film was initially positioned in the sagittal plane 2 cm away from the catheters, with the metal directly below and then 1 cm from the film. A uniform dose was delivered at the film plane. A second setup measured a depth dose curve in solid water with film in the transverse plane directly above the metal samples. This setup was recreated using Monte Carlo simulations (EGSnrc egs_brachy). Validation between methods was performed with unshielded (solid water only) measurements.

**Results:**

The planar dose passing through the metal samples (thickness 1–5 mm) at the midpoint between the film and catheters, decreased compared to solid water by 7.4% ± 6.9% to 26.5% ± 5.5%. Dose enhancement on the order of 5% was noted when metal was directly adjacent to the film. The average decrease in depth dose from a single dwell position ranged from 10.0% ± 5.9% (1 mm) to 21.1% ± 5.3% (5 mm) as measured with film, and from 3.8% ± 0.9% (1 mm) to 16.3% ± 0.9% (5 mm) using MC simulation. The average depth dose values were measured using a line width of 2.5 mm for film, and 3 mm for MC simulation, and the measurements generally agree within standard error.

**Conclusions:**

The 3D‐printed metal samples show potential for personalized applicators. Maximum dose reduction of 26.5% ± 5.5% compared to solid water was measured at 2 cm from the source using the 5 mm sample. An outer layer of solid water could potentially be used to reduce dose enhancement due to increased scatter near the metal.

## 1 INTRODUCTION

The use of 3D printing in gynecological brachytherapy is relatively new and can be used to improve both dose distribution and experience of patients.^1^, ^2^, ^3^, ^4^, ^5^ Non‐standard applicator geometries have been made to improve the fit to patients,^2^ while others are using 3D printing to modulate dose delivery.^1^, ^5^ Printing patient‐specific sizes and geometries of applicators allows for a more comfortable and accurate fit, particularly if the patient is between commercially available sizes. If the applicator is too small, it may slip or move between imaging and treatment, and if the applicator is too large, it could cause discomfort and other dose distribution difficulties.^2^ Printing a multichannel applicator also allows for customized channels along a patient‐specific shape.^6^ Another advantage of 3D printing an applicator is that interstitial guides can be included in the applicator design.^6^, ^7^, ^8^, ^9^

3D printing techniques can also be used to incorporate shielding into applicators, creating altered dose distributions. The effect 3D printing materials have on dose is not equal among different materials. Many plastics show water‐equivalency in the relevant energy region.^1^, ^2^, ^10^ Other work has used tungsten powder mixed with a plastic powder to print a high‐density object capable of dose shielding.^1^, ^5^, ^11^ The ability to combine water‐equivalent and shielding materials would allow the user to print an applicator with different sections capable of transmitting either a typical tumoricidal dose or a decreased shielded dose. A recently developed 3D printing technique that could facilitate such a design is Metal Jet printing.^8^ The metal powder is layered on the base plate and then a printing agent is used to bind the powder together in the shape designed for that layer of the object.^8^ Once all layers have been printed, the excess metal powder is removed from the surface and the object is then sintered to create a solid piece of metal.^8^ Once the finished object has cooled, any post‐processing finishing (e.g., smoothing, polishing) can be performed and the print is complete.^8^

Semeniuk et al. analyzed the shielding capabilities of many 3D printing materials using Monte Carlo simulations. All of the materials investigated are biocompatible and suitable for use in brachytherapy applicators.^1^ They had a range of densities and mass attenuation coefficients, but the printing plastics were similar for both metrics (1–1.32 g/cm^3^, and ∼0.1 mass attenuation coefficient).^1^ The stainless steels also showed similarities to each other, and the densest material investigated was a mixture of the printing plastic PLA and powdered tungsten (WPLA).^1^ Results showed the dose profiles in water using all materials in a generic applicator. The plastics were similar to the dose distribution in water, but the steels and WPLA provided 11% and 56% shielding, respectively, at 5 mm from the applicator surface.^1^

Pera et al. used film and an ionization chamber for the validation of 3D printed materials and associated attenuation compared to water using an Ir‐192 source. Frames for holding film were printed in the desired material, which was then submerged in water and irradiated exposing the film. Depth dose profiles were collected and compared to that of water.^12^ A custom phantom was also created to validate materials using an ionization chamber.^12^ The phantom was comprised of several boxes used to hold the material to be validated, the source, and the ionization chamber.^12^ The depth could be varied, and a depth dose curve was created with the data from the ionization chamber.^12^ The depth dose attenuation could then be compared with that of water.^12^ These methods both found that the materials tested were very similar to water (within 3%) and that the largest influence on dose variation was the distance from the source rather than the material in this phantom setup.^12^

The work described here aimed to characterize the shielding capability of 3D‐printed metal samples for potential use in personalized 3D‐printed shielded gynecological applicators. Quantification of the decrease in dose with the thickness of the metal would allow for future use of the 3D‐printed metals in personalized applicators. Using GAFchromic film and egs_brachy Monte Carlo simulations, the shielding capability was measured and compared.

## 2 METHODS AND MATERIALS

### 2.1 Film measurements

**FIGURE 1:**
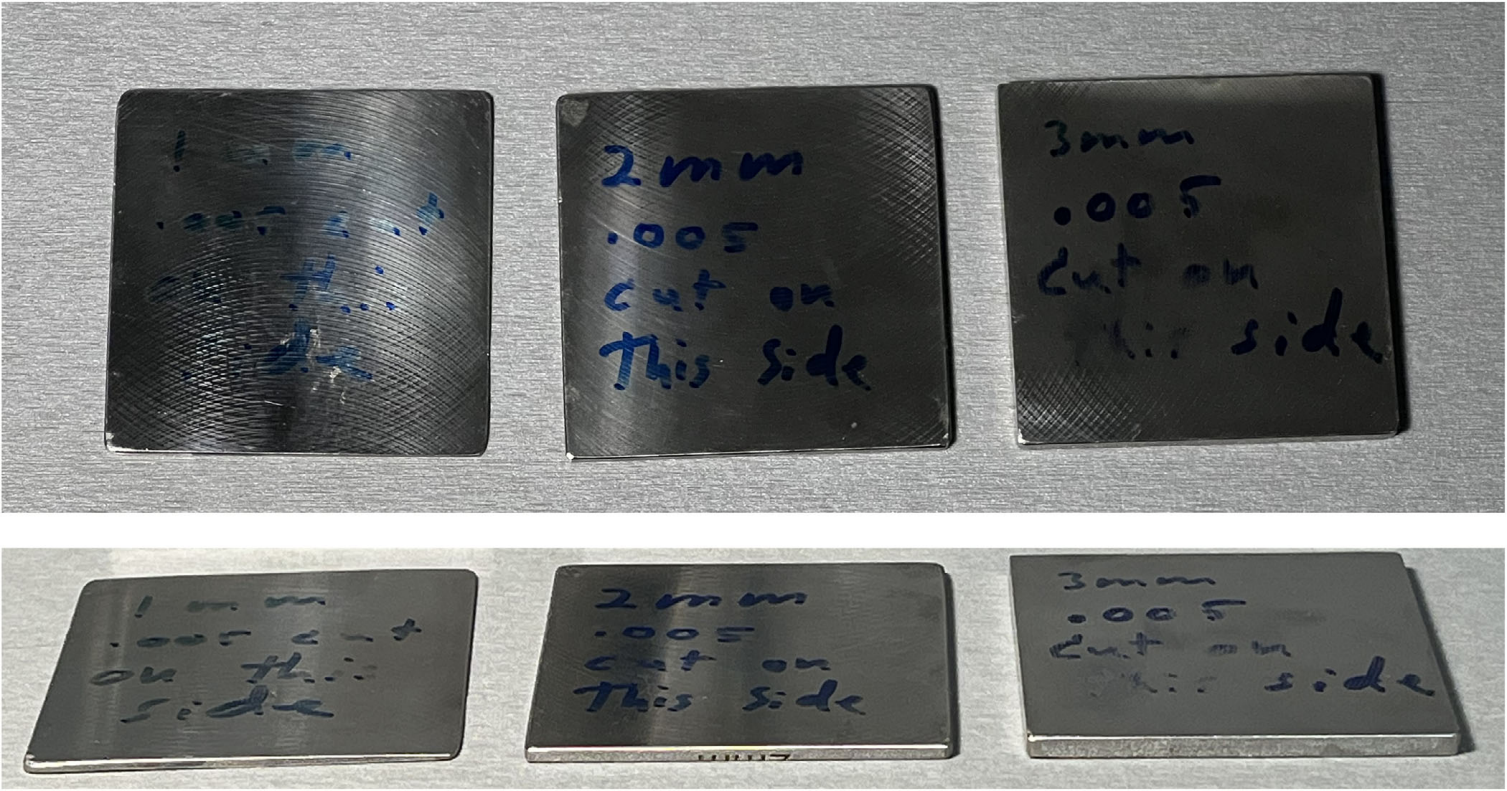
Metal samples (from left to right: 1, 2, and 3 mm).

Metal samples were provided by Hewlett Packard, HP, Palo Alto, California, in thicknesses ranging from 1–3 mm (Figure 1). They were all 3D printed with the same stainless steel. They were initially printed to a nominal thickness and then ground down to the various desired thicknesses. The average length and width of the samples were (54.4 ± 0.2) mm × (54.3 ± 0.2) mm. The density of the samples ranged from 6.7 to 7.9 g/cm^3^. Using Monte Carlo simulations, the density in the simulation was altered by ∼1 g/cm^3^ and this had a negligible effect on the results within error.

**FIGURE 2:**
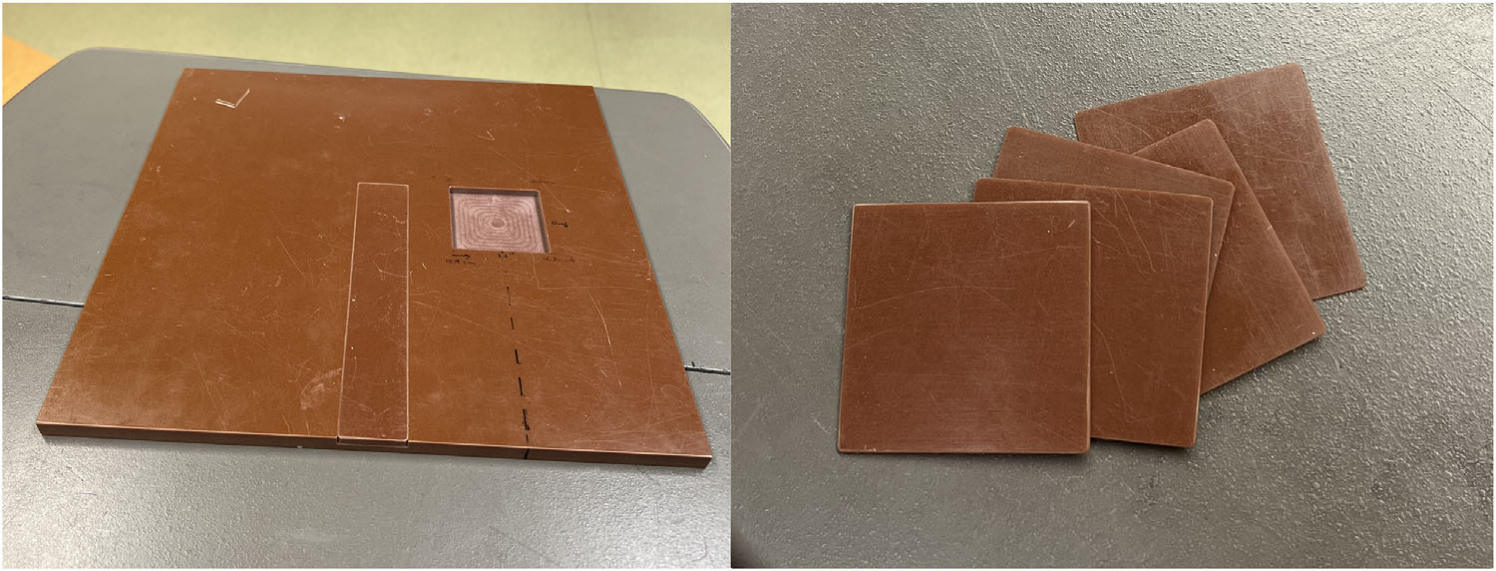
The custom water phantom with the hole for the metal samples and the five 1 mm pieces of solid water used to fill the air gaps.

A phantom made of solid water (Sun Nuclear Inc, Melbourne, FL, USA) with dimensions 30 cm × 30 cm × 1 cm was modified to insert the metal samples (Figure 2). An indentation was cut into the phantom with the dimensions of the samples (5.5 cm × 5.5 cm) and a thickness of 5 mm. A slab 1 mm thick of solid water was cut into pieces to fill the area cut out to eliminate air space when metal samples of smaller thickness were used. This phantom was used in all film measurements when the metal was in place.

The film used for all measurements was an EBT3 film (Lot 03082201) from Ashland (Wilmington, Delaware). The source used is a MICROSELECTRON V2 Ir‐192 source (Alpha‐Omega Services, Inc., Edgerly, LA) housed in an HDR remote afterloader (Nucletron, Elekta, Stockholm, Sweden).

#### 2.1.1 Calibration measurements

**FIGURE 3:**
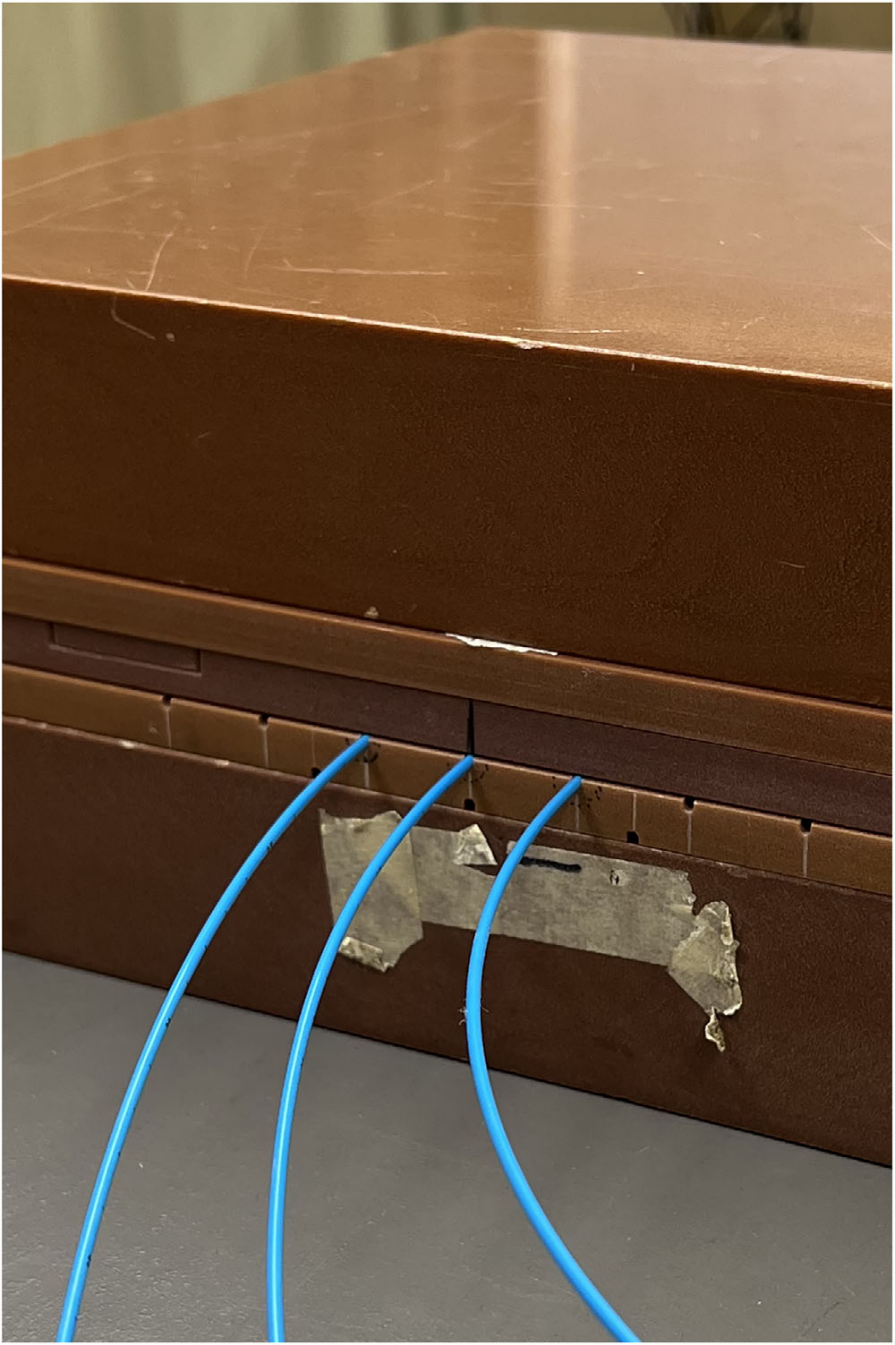
Photograph of the assembled planar phantom, catheters are blue and inserted into the phantom via the grooves.

To position the flexible catheters in the rigid solid water phantom, a 30 cm × 30 cm × 1 cm solid water phantom with catheter cutouts was used. It has seven grooves for holding the closed ends of catheters inside the phantom. The central three were used for the measurements. The catheters were placed, and masking tape was used to secure them, so they did not slip when adjusting the phantom arrangement between film exposures (Figure 3).

**FIGURE 4:**
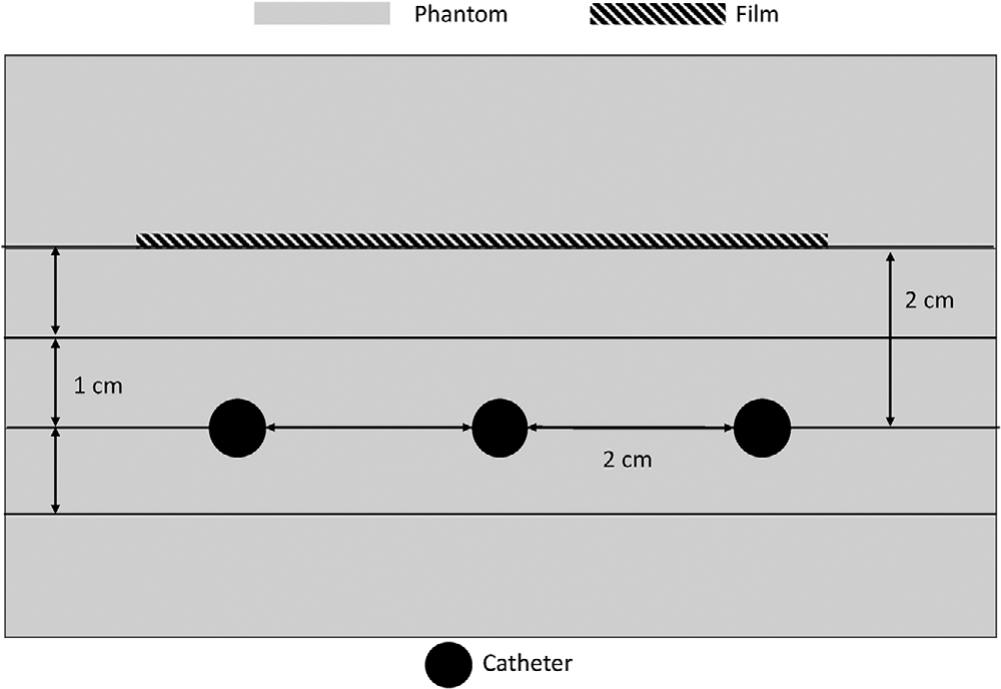
Graphic showing a transverse view of the calibration setup. Not to scale and only dimensions of film and catheter positions are shown. Additional scatter extending 5 cm in either direction was also in place for experiments, not displayed in the figure.

The film (which was cut into squares ∼7 cm^2^) was placed 2 cm from the plane of the catheters for all planar measurements. This 2 cm separation consisted of additional solid water. Tape was used to mark the measured distances from the dose delivery plan on the phantoms to quickly reference the distances to shorten set‐up time. Some test measurements were taken to ensure proper placement of the film along both axes. The film was placed above the dwell positions as well to capture the maximum dose reading. A 5 cm thick phantom was also placed above the film and below the slab with the catheters to provide adequate scatter conditions (Figure 4).

**FIGURE 5:**
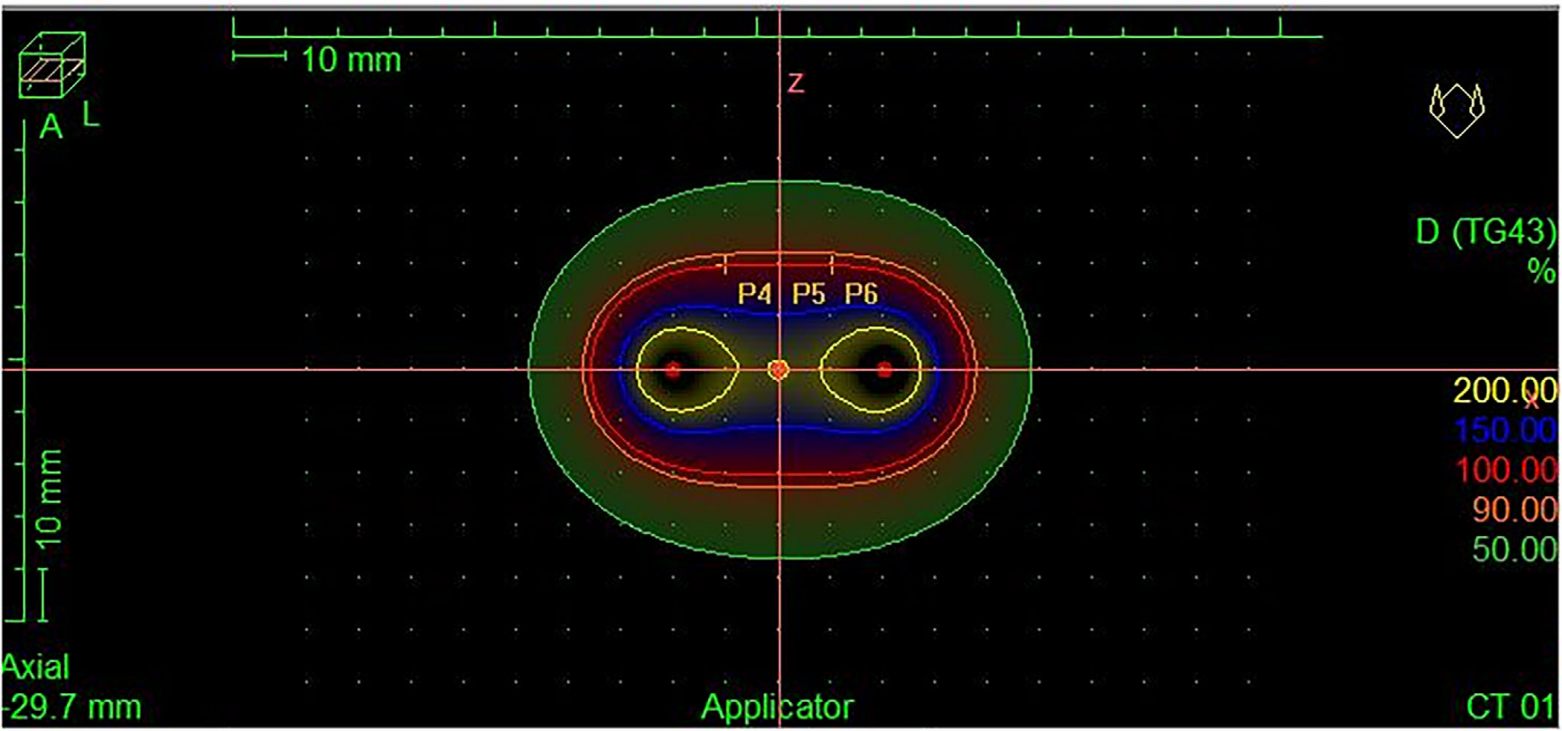
Oncentra view of the treatment plan for planar measurements. The dwell positions are highlighted with red dots along the blue catheters.

A treatment plan was created using Oncentra Brachy (Elekta, Stockholm, Sweden) to deliver a uniform dose across a region approximately 2 × 2 cm^2^ at a distance of 2 cm from the catheter. The same dwell positions and relative weights were used for calibration and planar dose measurements, with absolute dose and dwell times scaled to the appropriate values (see Figure 5). The film calibration curve required scans to be taken at even dose intervals. Measurements were taken for doses ranging from 0 to 400 cGy in intervals of 50 cGy. The films were scanned 3 days after exposure, to allow for self‐development as recommended by the manufacturer. They were then imported into the FilmQAPro software (Ashland, Delaware, US) and a calibration curve was created. The films for all measurements were scanned on a different day than the acquisition of calibration film. Recalibration (normalization) scans were then needed for each measurement day. These were taken at 0 and 400 cGy (with only solid water present).

#### 2.1.2 Planar measurements

**FIGURE 6:**
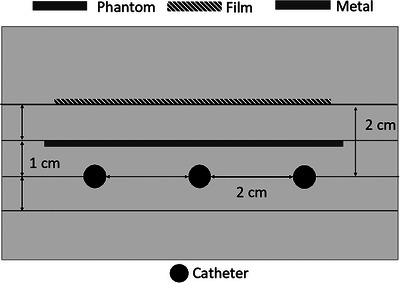
Experimental set‐up with metal sample halfway between the film and the catheters.

**FIGURE 7:**
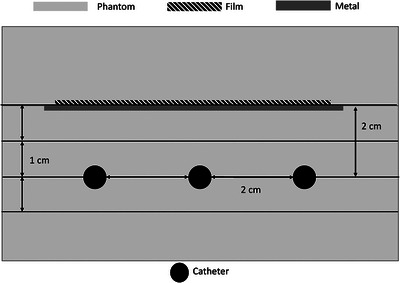
Graphic of the planar set up with metal sample directly adjacent to the film.

The planar measurements were taken using the same phantom orientation and film placement as the calibration set‐up. The measurements with the metal present were taken in two different arrangements. The custom phantom with the metal was initially placed so that the top of the metal was 1 cm from the catheters and 1 cm from the film (Figure 6). A second set of measurements was taken with the metal directly adjacent to the film, with the top of the metal 2 cm from the catheters (Figure 7). When the metal pieces were inserted into the custom phantom cut‐out, the extra 1 mm pieces of solid water were used to change the depth of the cut‐out to match the metal sample (i.e., using the 2 mm metal sample the cut‐out would have three 1 mm pieces of solid water followed by the metal placed on top).

The dwell positions for these measurements were all based on the same plan created in Oncentra Brachy (Elekta, Stockholm, Sweden). Using Oncentra Brachy, three catheters were placed with 2 cm of separation and dwell positions were specified every 0.5 cm along 4 cm of the catheter. The furthest dwell position was used so as to place the exposed area as near to the center of the phantom as possible. The measurements with the metal samples were all taken with a dose value of 200 ± 3 cGy delivered to a plane 2 cm from the catheters. This was verified by exposing a piece of test film without any metal present.

#### 2.1.3 Depth dose measurements

**FIGURE 8:**
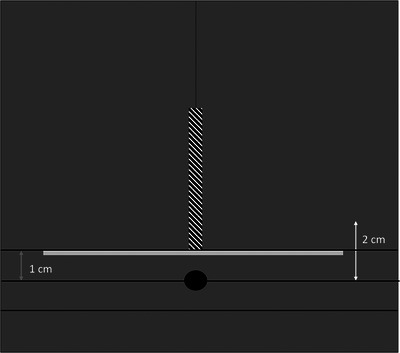
Graphic of the depth dose setup.

The depth dose measurements were taken with the film perpendicular to the plane of the catheters, to vary distance from the source. The base of the setup matches the design described earlier for planar measurements, with the 5 cm thick phantom underneath the phantom with the catheter cut‐outs. Above the phantom with the catheters was the custom phantom with the metal samples. The film was positioned vertically 1 cm from the source to capture dose fall‐off over the relevant region and to provide comparable results to the planar measurements. Imperfections near the edges of the film were present due to the preparation procedure. Measurements in the 5 mm closest to the source therefore contained inconsistencies in dose measurement and were excluded from analysis. The film was secured between two 30 cm × 30 cm × 5 cm solid water blocks, positioned with the 5 cm thick side down. Additional solid water blocks were placed on either side to achieve adequate scatter conditions (See Figure 8).

**FIGURE 9:**
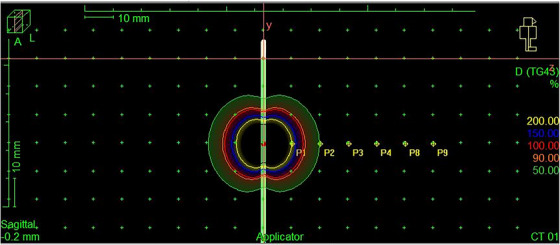
Oncentra view of the depth dose measurement treatment plan with the calculated dose values along a line perpendicular to the catheters. The dwell position is highlighted with a red dot in the central catheter (blue).

The depth dose measurements were taken using a single dwell position in the central catheter. This provided a simple geometry for a Monte Carlo model used to validate measured results. The dose was set to be 200 ± 3 cGy 1.5 cm from the source, as calculated in the Oncentra treatment planning system. The points measured in the planning software along the perpendicular direction were at 1 cm intervals as shown in Figure 9.

### 2.2 Egs_brachy modeling

**FIGURE 10:**
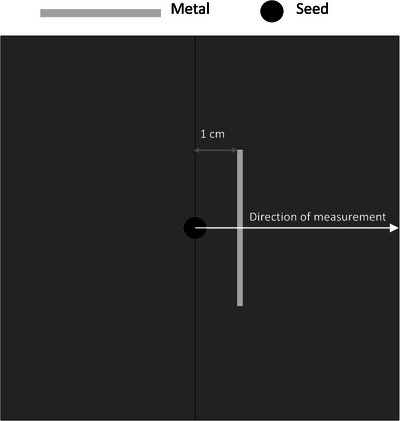
Graphic of the egs_brachy phantom.

The Monte Carlo package used to run the simulations was the egs_brachy package provided with the EGSnrc (National Research Council) code. The egs_brachy code is specific to brachytherapy simulations, and there are several pre‐configured materials, sources, geometries, and spectra that may be used for modeling. For this work, a simple custom geometry was created using water, the provided SS_AISI316L_p8.02 stainless steel material, and the Elekta MicroSelectron Ir‐192 source and spectrum. The geometry was contained in a 30 cm water cube, with a scoring region of a 10 cm cube of water with a 5 cm × 5 cm piece of steel with varying thickness. The steel was placed with the same geometry as the film measurements, with the furthest side of the metal placed 1 cm from the source (Figure 10).

**FIGURE 11:**
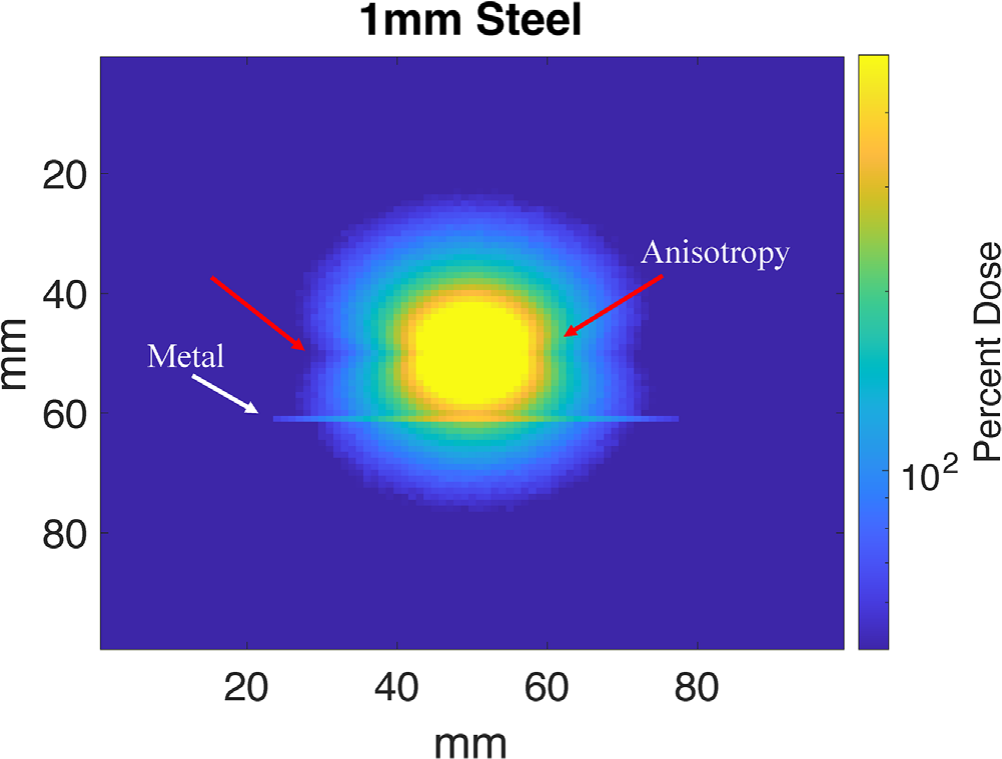
egs_brachy dose reading, showing the anisotropy of the single seed at the center (red arrows) relative to the plane of the metal (white arrow). The maximum value for the color scale is 500% dose.

The source was oriented so the anisotropy that occurs at the tip was not directed towards the metal (Figure 11). This matched the orientation used for the film‐based depth dose measurements. The simulation was run using $2\times 10^{8}$ histories and each particle was recycled 6 times to achieve an error <5% in the entire scoring area in a reasonable run time. Particle recycling is a standard function within egs_NRC used to increase the speed and decrease the variance of a particular calculation by reusing particles from the phase space. The dose deposition from the simulation was measured in the scoring region and was used for analysis.

## 3 RESULTS

### 3.1 Film

#### 3.1.1 Calibration curve

The calibration films were exposed to a dose range of 100 to 400 cGy in 50 cGy increments. The dataset also included an unexposed piece of film (0 cGy). The scanned images of the film were imported into the software FilmQAPro, which can be used to fit the calibration curves as well as analyze the dose from the film using the given calibration curve. All three calibration curves produced similar results, but due to the steeper slope in the red channel, it was chosen for continued calibration purposes. The steeper slope meant a greater sensitivity to changes in film density and corresponding dose values. To limit error due to the calibration process, only the red curve was used. It should be noted, however, that no major discrepancies were observed between the channels. Other work has used red channel calibration alone or in conjunction with other channels and it has been shown to be the most sensitive of the three color channels.^13^, ^14^, ^15^, ^16^

#### 3.1.2 Planar film measurements

**FIGURE 12:**
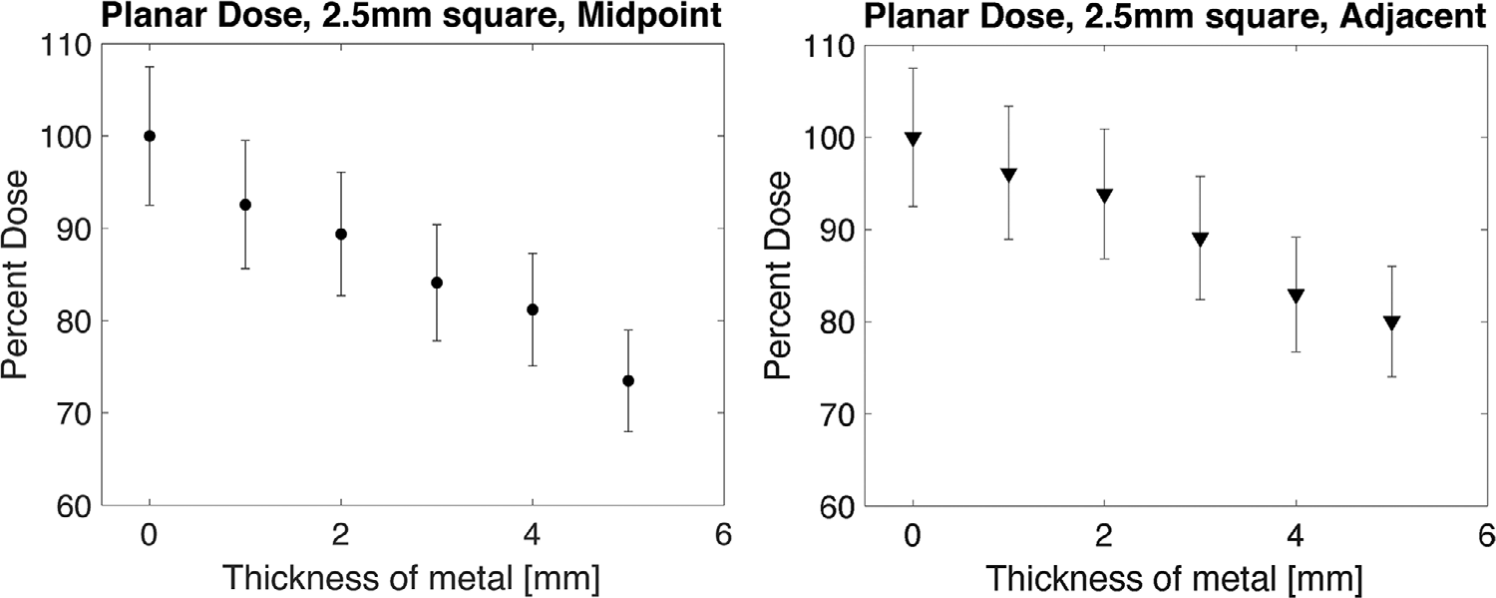
Planar percent dose delivered in water, measured using the red calibration channel. The values using the setup with the metal halfway between the catheters and the film are shown with a circle, and the values from the setup where the metal is directly adjacent to the film are shown with a triangle. The error displayed is a 7.5% error on the film measurement, detailed in Table 1.

A planned dose of 200 ± 3 cGy, delivered to the film in only solid water, resulted in a measured dose of 202.5 cGy which is within the expected range of dose output. The rest of the film samples were then read and normalized to the dose measurement in water alone. Uncertainty in film measurements was calculated based on methods described by Oare et al, detailed in Table 1. The error bars displayed in Figure 12 show the total calculated uncertainty of 7.5% for planar measurements. Results are shown below in Figure 12, with a minimum of 3.9% ± 7.1% shielding (1 mm of metal directly adjacent to the film) and a maximum of 26.5% ± 5.5% shielding (5 mm of metal at the midpoint). The average increase in dose due to scattered electrons from the metal in the adjacent measurements is 5.2%. The values were measured in a 2.5 mm square on the film, centered on the pixel with the highest dose value.

#### 3.1.3 Depth dose

**FIGURE 13:**
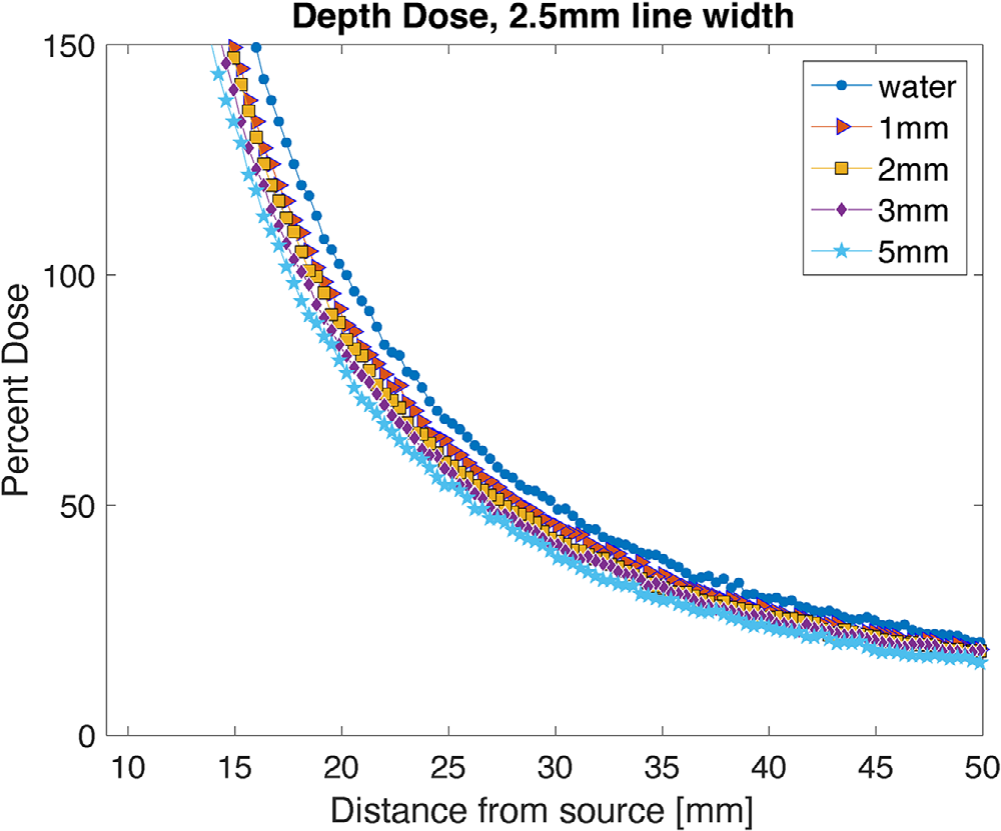
The depth dose measured with film using varying thicknesses of metal ranging from 0 to 5 mm.

**FIGURE 14:**
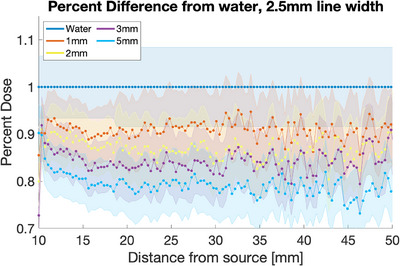
The percent difference from water measured using film. The metal thicknesses range from 1 to 5 mm. The error from the film measurements is shown as the associated shaded region for each color/thickness of the sample.

These films were scanned in the same batch some time after the creation of the calibration curve, so they required calibration curve reference films for recalibration. To allow for the recalibration, films were taken at 0, 200, and 400 cGy, in the planar orientation without any metal present. The 0 and 400 cGy films were used to recalibrate the calibration curve, and the 200 cGy film was used to test the accuracy of the recalibration. To find the area on the film to capture the depth dose measurement, the maximum value 15 pixels from the edge of the film was found and then a width of 2.5 cm (7 pixels) was averaged to create the depth dose data. The dose delivered in water only was used to normalize the film, and the point at 2 cm from the source was set to 100% dose. Due to separation at the edge of the film which occurs during preparation, the first 10 mm were disregarded. To better visualize the shape of the curves, values are plotted to a limit of 150% (Figure 13). The relative dose difference is also plotted along the depth and shows a maximum of 21.1 ± 5.3% shielding (Figure 14).

**FIGURE 15:**
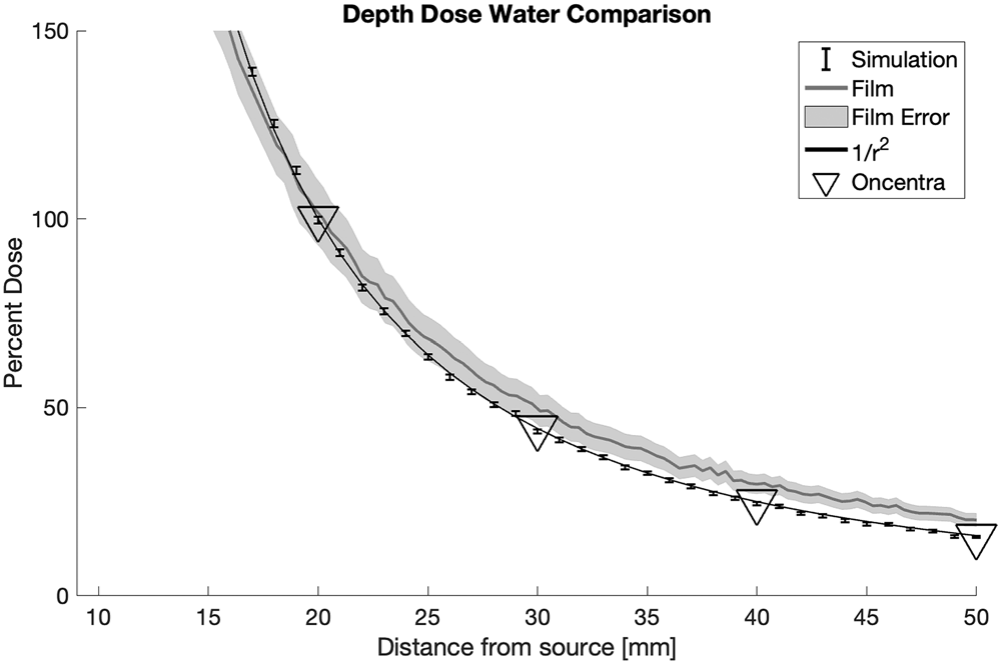
A comparison of different depth doses in water using egs_brachy, film, the Oncentra planning system, and geometric $1/r^{2}$ falloff from a point source. The shaded area around the film curve shows the error of the film measurements.

To determine if the simulation and film results were behaving as expected, the depth dose curves measured without metal were compared to (i) $1/r^{2}$ geometric fall off of dispersion in a vacuum from a point source and (ii) dose values calculated from the depth dose plan in Oncentra as displayed in Figure 9. The shape of all of the depth dose curves agree within error, confirming the absence of any gross error (Figure 15).

### 3.2 Egs_brachy

**FIGURE 16:**
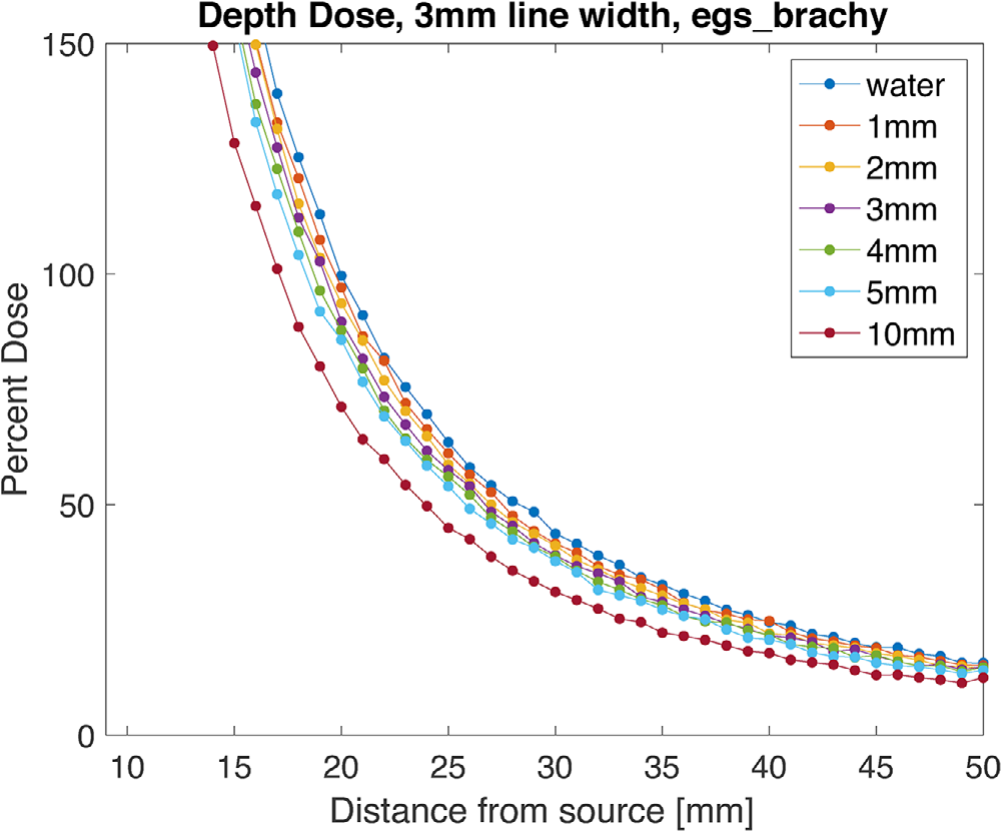
Depth dose measured using egs_brachy simulations. The thickness of metal simulated ranges from 1 to 5 mm and includes 10 mm as a projection.

**FIGURE 17:**
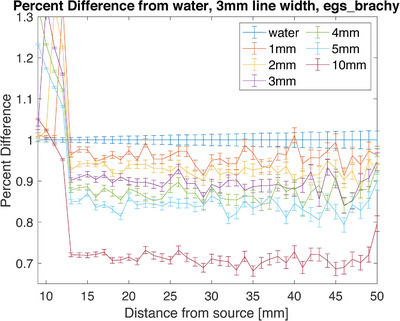
Percent difference measured using egs_brachy simulations. The metal thicknesses range from 1 to 5 mm and includes 10 mm as a projection.

Using egs_brachy to simulate a single Ir‐192 seed in the presence of varying thicknesses of metal, the following data was collected. The region around 10 mm shows some dose enhancement due to its proximity to the steel. To find the area of the produced data to average for the depth dose, the central pixel and the two pixels on either side were averaged (3 pixels are 3 mm wide). The point 2 cm from the source when only water was present was used as the normalization point and was set to 100% dose (Figure 16). Maximum shielding was seen for the 5 mm thick sample, which provided a decrease in dose of 16.3 ± 0.9% 2 cm from the source. For theoretical purposes, a 10 mm sample of steel was also modeled, which provided approximately 30% shielding at the same point (Figure 17).

## 4 DISCUSSION

### 4.1 Uncertainty analysis

The uncertainty for the egs_brachy simulation is determined from the parameters of the simulation. The uncertainty analysis for the film is taken from Oare et al, which is influenced by the method suggested in TG‐43. The intention of Oare et al. was to determine any dose differences and uncertainties for 3D printed water equivalent applicators for brachytherapy. The underlined descriptions in Table 1 are the same values as in Oare et al., since the same type of film and source were used. The ROI size used in this work is included in the range for 1.5% error.^14^ The distance to source error was calculated using the $1/r^{2}$ principle using a 1 mm shift for the planar dose measurements and a 2 mm shift for the depth dose measurement.^14^ Due to possible positioning errors, a 2 mm shift was chosen for the depth dose measurement error, and due to the potential variation in metal thickness which could leave air gaps, an uncertainty of 1 mm was chosen for planar dose measurements. The exposure time used the same error of 0.4 s from Oare et al. but scaled for delivery for 200 cGy exposure (∼90 s).^14^ The calibration curve fit comes from the discrepancy between 200 cGy and the test piece of film used to check the corrected calibration used for dosimetry. All the film had a 3% error on the corrected calibration. The total errors in the table below show the error displayed in the film measurements in the shaded regions of the figures.

**TABLE 1:** Description and value of uncertainties, using the method of Oare et al. The underlined descriptions are taken directly from Oare et al.

Uncertainty description	Value
Scanning consistency	0.1 %
Film uniformity	1.0 %
ROI size	1.5 %
Calibration curve fit	3.0 %, 6.0% (low dose, depth dose only)
Repeatability	0.1 %
Distance to source	4.0 % (planar), 2.0% (depth dose)
Lack of scatter equilibrium	5.0%
Source strength	1.5%
Exposure time	0.4%
Total	7.5% (planar), 6.6% (depth dose), 8.4% (depth dose, low energy)

### 4.2 Planar measurements

The planar dose delivery included several different seed positions and path lengths traveling through the metal. This is most similar to how a patient would be treated (instead of a single seed position). As the thickness of metal increases, the dose deposited past the metal decreases. This aligns with expected results based on $1/r^{2}$ predictions. There is also an increase in dose when measurements were taken directly adjacent to the metal, implying that there are some dose enhancement effects present when the metal is irradiated. This is not an unexpected outcome, as when metal is irradiated some electrons are produced, due to the photoelectric effect and Compton scattering, which would increase the dose in the most immediate tissue.^17^

The decrease in dose as the thickness increases is generally consistent, with an average decrease of 5.3% dose/mm of steel for the planar measurements taken 2 cm from the plane of the catheters. The planar measurements are an average of a 2.5 mm square centered on the maximum pixel value found in the area of dose delivery. There are some larger differences, namely from 0 to 1 mm, and 4 to 5 mm when the metal was midway between the catheters and the film. These had an average dose decrease of 7.5%. The same fluctuation is not seen in the measurements from the film that is directly adjacent to the metal, and these values decreased an average of 4.0% dose/mm of steel.

The 5 cm × 5 cm × 5 mm piece of steel weighed 116 g, which when compared to the same volume of water (12.5 g) is a considerable increase in weight. Depending on the size of the area that is intended to be shielded, there may be even more steel present in a 3D‐printed applicator. This may affect the comfort and ease of use of the applicator, while only providing between 20% and 26% shielding. The effectiveness of this specific material may not be ideal for total OAR shielding but would be able to provide partial shielding or dose modulation. The rectum, bladder, urethra, colon, sigmoid, vaginal wall, and mucosa are nearby organs that are considered when creating treatment plans for vaginal brachytherapy.^1^, ^5^, ^18^, ^19^, ^20^ One work presented the mean dose to some of these organs using a conventional single‐channel cylinder.^21^ The doses were: bladder 23.7% ± 8.8% and 35.7% ± 13.3%, rectum 24.6% ± 5.6% and 37.2% ± 8.6%, sigmoid 15.2% ± 7.6% and 24.2% ± 12.3%, urethra 24.1% ± 12.2%, and 36.4% ± 16.5%, as percent prescription dose at the surface of the applicator and a 0.5 cm depth from the applicator surface.^21^ This was achieved with a water equivalent applicator, so the presence of shielding using the same dose delivery plan could allow for reduced doses to these nearby organs while providing the same dose to the target volume. Using the same treatment plan, the inclusion of steel could decrease the dose to these organs further but would not be able to provide full shielding.

The Semeniuk et al. work investigated theoretical 3D‐printed gynecological brachytherapy designs based on the applicator described in TG‐186. The applicator has a diameter of 3.6 cm, with a central channel diameter of 3.2 mm (central 1 mm is an air channel for a catheter and the surrounding 2.2 mm are made of stainless steel).^1^ One of the materials investigated was the same stainless steel used in this work. This leaves a maximum of 1.64 cm for shielding material thickness. The Skinner et al. work uses an applicator design that is 30 mm in diameter and features a Miami style applicator with a central catheter channel and more channels distributed radially. This design allows for a maximum thickness of less than 15 mm, which is consistent with Semeniuk et al.

Extrapolating from Figure 12, 15 mm of stainless steel would provide approximately 70% shielding. Semeniuk et al. find that WPLA is able to provide approximately 80% shielding. Skinner et al. find that using a Miami style applicator, they are able to achieve between 40% and 50% shielding, but they intend to print applicators using the denser material WPLA. WPLA has a density of 9 g/cm^3^ and the stainless steel used in this work has a density of approximately 7.2 g/cm^3^.WPLA, however, has a much higher mass attenuation coefficient (WPLA 0.2130 cm^2^/g, stainless steel 0.0979 cm^2^/g for 380 keV photons).^1^

### 4.3 Depth dose

Cunha et al. showed that film could be used to determine water equivalency of a material. A custom apparatus was printed using PC‐ISO and was printed to hold a piece of film and a catheter.^2^ The film was positioned so it was perpendicular to the catheter and able to capture the depth dose measurement through PC‐ISO.^2^ The dose plan used to deliver the radiation had dwell positions along the length of the apparatus.^2^ The film was exposed in the PC‐ISO apparatus and while it was submerged in water, the results were compared and were found to be within 1% of each other.^2^

The water measurements from Figure 15 show that the simulation and film measurements are comparable, at least until 40 mm from the source. Oare et al. discuss the error found when using film for dosimetry, and that the error increase for EBT3 film at low doses primarily due to a decreased signal‐to‐noise ratio, which they determined was 100 cGy and below.^13^ The film used reaches 100 cGy between 25 and 30 mm from the source, depending on the thickness of the metal present. Therefore, the error values increase past this distance to account for the accuracy of the film at lower doses.^14^

The simulation and film results agree within error (simulation 16.3% ± 0.9% and film 21.1% ± 5.3%, for 5 mm of steel), and both show the expected increase in shielding as the thickness increases. The steel samples used in the MC model had a density of 8.02 g/cm^3^ which was slightly higher than the measured density of the metal samples used in film experiments (average density 7.2 g/cm^3^). This may cause a slight systemic difference between the measurements, but the uncertainty of the measurements supersedes any slight difference. They also show that the shielding is maintained as the distance from the source increases. These results show nominal shielding capabilities to be lower than the planar results but appear to more closely agree with the planar measurements taken directly adjacent to the metal (20.0% ± 6.0% adjacent for 5 mm of steel). A Wilcox‐Rank test was performed on the two data sets and found that there was a difference between the two, however, it was not statistically significant. Since the depth dose measurements are also taken with direct contact with the metal, this shows the consistency of potential dose enhancement between the measurement methods. Using Monte Carlo simulations, a projection at 10 mm thickness was calculated and resulted in a predicted 30% shielding to be achieved with that thickness.

Semeniuk et al. and Skinner et al. both use multiple source dwell positions. Due to the range of angles through the shielding material that occurs with multiple dwell positions, the magnitude of shielding will not necessarily match the shielding that is measured from a single dwell position. This causes the shielding capabilities measured with multiple dwell positions to be difficult to compare with shielding measured with only one dwell position because the radiation conditions and paths through the shielding conditions differ.

## 5 CONCLUSION

This work sought to characterize the shielding capabilities of stainless‐steel samples as a means for brachytherapy treatment with 3D‐printed applicators. Radiochromic film and egs_brachy simulations were used to measure the shielding capabilities of a range of metal thicknesses (1 to 5 mm). The 5 mm of steel in the planar orientation was found to provide 26.5% ± 5.5% shielding when the metal was 1 cm away from the film, and 20.0% ± 6% shielding when the metal was directly adjacent to the film. The 5 mm steel sample in the depth dose orientation provided 21.1% ± 5.3% shielding when measured with film and 16.3% ± 0.9% shielding when simulated using egs_brachy. An increase in thickness to 10 mm increases the shielding to ∼30% when simulated. The stainless steel is able to provide shielding, but the shielding values are not significant when considering the additional time and workload needed to produce a personalized applicator. To improve the shielding capabilities, more metal would need to be used and the thickness is limited to the size of the applicator being used.

A continuation of this work could include the characterization of other high‐density 3D printing materials, testing printed or simulated applicators, and optimizing the process of determining the areas to be shielded.

## AUTHOR CONTRIBUTIONS

K. Maiti McGrath and Amanda Cherpak contributed to the data acquisition and analysis, and all authors contributed to the design of the work and interpretation of the data. K. Maiti McGrath drafted the manuscript, and all authors critically reviewed and revised it, and approved the final version to be published.

## CONFLICT OF INTEREST STATEMENT

The authors declare no conflicts of interest.
